# Tumor Mutational Burden Associated With Response to Hyperthermic Intraperitoneal Chemotherapy

**DOI:** 10.3389/fonc.2022.796263

**Published:** 2022-03-08

**Authors:** Lisi Zeng, Xubo Huang, Yun Tian, Jinxia Huang, Huiyan Liu, Juncai Wen, Kaihua Liu, Yang Shao, Jiali Luo, Hongsheng Tang, Quanxing Liao, Ziying Lei, Weiwen Cui, Qianghua Xia, Tianpei Guan, Jin Li, Shuzhong Cui

**Affiliations:** ^1^ Affiliated Cancer Hospital and Institute of Guangzhou Medical University, Guangzhou, China; ^2^ Department of Abdominal Surgery, Affiliated Cancer Hospital & Institute of Guangzhou Medical University, Guangzhou, China; ^3^ Medical Department, Nanjing Geneseeq Technology Inc., Nanjing, China; ^4^ Department of Bioengineering, University of California, Berkeley, Berkeley, CA, United States; ^5^ State Key Laboratory of Respiratory Disease, Guangzhou Medical University, Guangzhou, China

**Keywords:** gastric cancer, hyperthermic intraperitoneal chemotherapy, survival, tumor mutational burden, biomarker

## Abstract

**Background:**

Gastric cancer (GC) is one of the most common cancer types, especially in Asian countries. Hyperthermic intraperitoneal chemotherapy (HIPEC) has been shown to improve the progression-free survival among gastric cancer patients with peritoneal metastases; however, not all patients demonstrate response to HIPEC.

**Methods:**

Biomarkers are needed to select patients for effective treatment of HIPEC. Here, we performed whole-exome sequencing on tumor samples from 18 gastric cancer patients who received HIPEC treatment and assessed the association between genomic mutation features and progression-free survival. Exome sequencing was further conducted on tumor samples from additional 15 gastric cancer patients as a replication study.

**Results:**

The tumor mutational burden (TMB) was significantly higher in the group of patients with a better response to HIPEC treatment than that of the others. Kaplan–Meier survival curve showed that patients with high TMB had a significantly longer survival time than that in patients with low TMB. This discovery was validated in the replication cohort. Genes bearing mutations recurrently and selectively in patients with better response to HIPEC were found in the two cohorts.

**Conclusion:**

We found that higher TMB is significantly associated with better response to HIPEC. Our results provide useful hints for prognostic stratification of HIPEC treatment.

## Introduction

Gastric cancer is among the most common cancer types and a leading cause of cancer death in both men and women worldwide (1), especially in Asian countries, which imposes a considerable global health burden. In addition, when gastric cancer is diagnosed, patients are often already at an advanced stage. Surgery with subtotal gastrectomy or total gastrectomy is the current mainstay of treatment (1). Surgical resection is a curative therapeutic approach for gastric cancer and confers good outcome for early-stage gastric cancer. However, early gastric cancer often remains asymptomatic unless detected by endoscopy and biopsy. The survival rate for patients with metastatic gastric cancer is very low, ranging from 4 to 12 months (1), despite the successful application of modern chemotherapy to other solid tumors.

Hyperthermic intraperitoneal chemotherapy (HIPEC) is carried out by perfusing the abdominal cavity with 43°C circulating hyperthermic saline containing anticancer drugs. HIPEC treatment has been applied to gastric cancer, colorectal cancer, ovarian cancer, pancreatic cancer, and other abdominal cancer types. Poor peritoneal penetration of chemotherapy drugs is an outstanding limitation in the treatment of gastric cancer. HIPEC has thus been administered to patients in combination with cytoreductive surgery to achieve better therapeutic effects. Cumulative evidence has shown the beneficial treatment effect of HIPEC. Studies showed that a combination of cytoreductive surgery (CRS) and HIPEC can reduce the incidence of peritoneal recurrence of advanced gastric cancer and improve the median survival (2–4). A systematic review (5) on 7 studies (6–12) has been done to compare the prophylactic HIPEC after surgery for patients with gastric cancer without clinically evident metastases or positive peritoneal cytology sign. Their analysis results suggested that, compared to surgery alone, the combination with HIPEC may decrease peritoneal recurrence and increase survival rate without affecting the morbidity and mortality of patients despite the overall risk of bias in these studies that is likely due to the non-standard of care in the studies that was carried out more than 10 years ago. A meta-analysis of randomized controlled trials in patients with advanced gastric cancer and peritoneal metastases also showed a beneficial effect of HIPEC in terms of 3-year survival rate and complete response rate (13). Continuous breakthroughs have been made in developing the theoretical basis and technical execution in HIPEC, which further improved the treatment effect (14, 15). Because of the promising treatment of HIPEC, the International Conference on Peritoneal Cancer in Amsterdam (Netherlands) and the American Anti-Cancer Association adopted CRS combined with HIPEC as the standard treatment for gastric cancer with peritoneal metastasis (16, 17).

Similar to the fact that cancer patients respond distinctly to immunotherapy, patients also have different responses to HIPEC treatment. The different results observed regarding the efficacy of HIPEC imply that not all patients can benefit from the treatment and proper stratification may be necessary before HIPEC treatment (14, 15). Few studies have been carried out to examine the biomarkers that can effectively predict the efficacy of HIPEC and stratify patients for HIPEC treatment. Through a candidate gene approach, three studies identified biomarkers, the expression level of which can be predictive for the efficacy or resistance to HIPEC treatment on patients with colorectal peritoneal metastasis, including Bloom syndrome protein (BLM) (18), Vascular endothelial growth factor (VEGF), Versican (VCAN) (19), PAX interacting protein 1 (PAXIP1), and Single stranded DNA binding protein 2 (SSBP2) (20). A recent study focusing on candidate genetic variants reported the association of NQO1∗3 allele with poor peritoneal recurrence rate and low disease-free survival (21) among colorectal cancer patients who received cytoreductive surgery plus hyperthermic intraperitoneal mitomycin C. There was no study examining the genetic association with efficacy of HIPEC on gastric cancer. Furthermore, an unbiased study *via* a genomic approach may be more fruitful in identifying biomarkers to predict HIPEC efficacy.

## Methods

### Tumor Samples and Clinical Characteristics

The study was approved by the institutional review boards of the Affiliated Cancer Hospital of Guangzhou Medical University. All tissue samples were obtained with the approval of patients’ consents from 2010 to 2017. The recruited patients met the following criteria. They had proven gastric cancer with histopathology and received CRS and closed HIPEC treatment after surgery. HIPEC was administered intraperitoneally with chemotherapeutic agents in 4–6 L of perfusate at a temperature of 43°C for 90 min with a flow rate of 400–600 ml/min. The resected tumor samples were examined by the pathology department to confirm the stage of tumor tissue. The tumor tissues and adjacent regions were prepared as formalin-fixed paraffin-embedded (FFPE) tissue blocks following the standard protocol of the diagnosis laboratory.

Approximately 30% of GC patients have regional spread at diagnosis; the local regional progression of gastric cancer generally results in peritoneal metastases (PMs), which have a significant negative impact on the overall survival (OS) and quality of life as a result of refractory ascites, progressive intestinal obstruction, and uncontrollable abdominal pain (22). CRS+HIPEC could obviously decrease the volumes of ascites for a long time compared to only CRS for advanced GC patients. We checked the OS as the criteria of HIPEC effect. Patients with survival time of more than 1 year after HIPEC treatment were classified as the durable clinical benefit (DCB) group, and those with less than 1 year were included in the no durable benefit (NDB) group.

### Statistical Analysis

The data analysis was conducted using IBM SPSS Statistics 24.0 (SPSS, Inc., Chicago, IL, USA). Continuous data were expressed as mean ± standard deviation (SD), and inter-group comparison was conducted using an independent-samples t-test. Count data were expressed by percentage or constituent ratio, and the comparison between groups was carried out by chi-square test or exact probabilities method. The level of statistical significance was set at p < 0.05 and α = 0.05. Specifically, the comparison of baseline characteristics of patients was conducted with the following statistical methods: age between groups was examined by unpaired t test; the comparison of sex, Eastern Cooperative Oncology Group (ECOG), and degree of differentiation between groups was examined by chi-square test; and the comparison of the degree of peritoneal metastasis (P degree), ascites, and number of chemotherapy in half a year between groups was examined by Fisher exact test. The Kaplan–Meier (K-M) method and the log-rank test were used to evaluate OS.

### Whole-Exome Sequencing

Tumor samples and para-cancer control samples from FFPE tissue blocks were analyzed by whole-exome sequencing (WES). The para-cancer control samples were taken from the normal tissue within the 1–5-cm distance from the visible tumor area. Library preparations were performed with KAPA Hyper Prep Kit (KAPA Biosystems, USA). Target enrichment was performed using the xGen Exome Research Panel and Hybridization and Wash Reagents Kit (Integrated DNA Technology, USA) according to manufacturer’s protocol. Standard WES was performed with paired-end sequencing on the Illumina HiSeq2000 platform to generate reads of 2 × 100 bp with an average of 200× mean target coverage for tumor samples and 20× mean coverage for controls.

### Exome Analysis Pipeline

Quality control and filtering steps were performed on the raw sequencing data, and the data were further checked using software FastQC, including per sequence quality scores, GC content, per base sequence quality, per base sequence content, sequence duplication levels, and overrepresented sequence. Then, the Quality Control (QC)’ed sequencing data were aligned to the Grch37 genome built using Burrows–Wheeler Aligner (BWA) (v0.7.12) (23). Picard Tool was used to mark duplicates, and Genome Analysis Tool Kit (GATK) was used to conduct indel realignment, base-quality score recalibration, and duplicate-read removal (24). Variant calls for both Single Nucleotide Variation (SNV) and Insertion Deletion (INDEL) were generated using software VarDict (25) based on the paired tumor–normal variant calling algorithm. Variant annotation was performed using ANNOVAR (26) and vcf2maf on the VCF file, and further filtering was described below.

### Variant Filtering

Variants were filtered out in any of the following conditions: (allele frequency × read depth <6) and (mean number of mismatches >1.0 and mean mapping quality <55.0); or (mean number of mismatches >2.0 and mean mapping quality <60.0); or (read depth <10) and (alternate allele quality <45); or alternate allele quality <55 and allele frequency <0.2 and somatic variant p-value >0.06. In addition, variants were filtered out if the reference genotype likelihood of their para-cancer control were >3.5. To keep stringent data quality, we only kept strong somatic variants. Then, variants were further filtered based on their predicted effect on protein structure and function and variant allele frequency in large population databases. Variants that might have an impact on protein function (e.g., in_frame_del, in frame_ins, missense_mutation, nonsense_mutation, nonstop_mutation, splice_site, and translation_start_site) and allele frequency <0.01 in the following databases were kept, including ExAC_EAS, gnomAD_exom_ALL, gnomAD_exom_EAS, gnomAD_genome_ALL, gnomAD_genome_EAS, 1000g2015aug_all, and 1000g2015aug_eas.

### Analysis of Tumor Mutational Burden on HIPEC Efficacy

Tumor mutational burden (TMB) was defined as the total number of strong somatic non-synonymous mutations in each tumor exome that passed our QC filtering. Mann–Whitney test was used to compare the number of somatic mutations in DCB and NDB groups. Patients were divided into the high mutation group and the low mutation group based on the median of somatic mutation number of all patients, which is 373. It is about 9.8 mutations/Mb, which is similar to the TMB threshold adopted in other studies (27, 28). Log-rank test was used to compare K-M survival curves between the two groups.

### Association Analysis at Single-Variant Level

We first evaluated the association of variants with HIPEC efficacy at the single-variant level by Fisher’s exact test, and the statistical significance threshold was p-value <0.05.

### Association Analysis at the Gene Level

RVTESTS (29) were used to perform correlation analysis of rare variants at the gene level. We used the VCF file generated from the above pipeline as the input file for RVTESTS, followed by Fisher’s exact test (CMC Fisher) to evaluate genes being positively or negatively associated with the efficacy of HIPEC.

### Pathway Enrichment Analysis of Genes Associated With HIPEC Efficacy

Pathway enrichment analysis was performed *via* WEB-based GEne SeT AnaLysis Toolkit (30) (http://www.webgestalt.org/) that is based on the hypergeometric test.

From International Cancer Genome Consortium (ICGC) databases (https://dcc.icgc.org/), whole-genome sequencing data and follow-up data of Chinese population were obtained, and the TMB value of non-synonymous mutations in the exon regions was calculated for each patient. The patients were divided into high TMB group and low TMB group by the median value of TMB, and K-M survival curve was performed to examine the association between TMB and survival.

## Results

### High Tumor Mutational Burden Was Associated With Improved Patient Survival

We conducted a retrospective study to identify genetic determinants for HIPEC response involving 18 gastric cancer patients, among whom 8 patients showed DCB (patients with survival time of more than 1 year) and 10 patients had NDB. The baseline characteristics of these patients are shown in **Table 1**. The average age of the NDB group is significantly lower than that of the DCB group (43.8 ± 9.7 vs. 54.1 ± 8.1, p = 0.030), while the other characteristics were similar between DCB and NDB groups. We analyzed the WES data of these tumor samples with their matched normal tissues for germline references. The mean target coverage in the WES data of tumor samples is 200× and, on average, >90% of the target sequence was covered to a depth of >10×. The number of non-synonymous somatic mutations in all patients ranged from 90 to 3,027, with a median of 373.

**Table 1:** The baseline characteristics of patients who received hyperthermic intraperitoneal chemotherapy (HIPEC).

	DCB (*n* = 8)	NDB (*n* = 10)	P
Age	54.1 ± 8.1	43.8 ± 9.7	0.030*
Sex			
Male	3 (37.5)	4 (40.0)	0.999
Female	5 (62.5)	6 (60.0)	
ECOG			
0~1 score	7 (87.5)	9 (90.0)	0.999
2~4 score	1 (12.5)	1 (10.0)	
Degree of differentiation			
G1+G2	2 (25.0)	2 (20.0)	0.999
G3+GX	6 (75.0)	8 (80.0)	
P degree			
p1x	1 (12.5)	0 (0.0)	0.999
p1	1 (12.5)	2 (20.0)	
p2	3 (37.5)	4 (40.0)	
p3	3 (37.5)	4 (40.0)	
Ascites			0.552
No	6 (75.0)	5 (50.0)	
A small amount number of chemotherapy in half a year	2 (25.0)	5 (50.0)	
			
1~3	7 (87.5)	7 (70.0)	0.751
4~6	1 (12.5)	3 (30.0)	

ECOG, the grade of Eastern Cooperative Oncology Group. They were classified into durable clinical benefit (DCB) group and no durable benefit (NDB) group based on their response to treatment. *p-value < 0.05.

**Figure 1:**
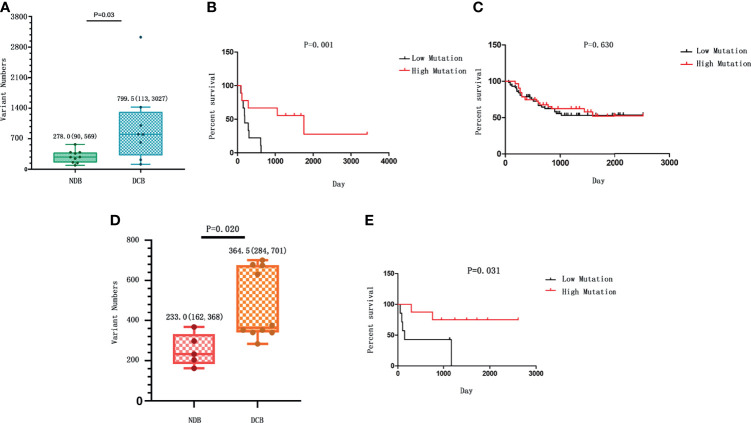
The correlation between tumor mutational burden (TMB) and response to hyperthermic intraperitoneal chemotherapy (HIPEC). **(A)** The number of strong somatic mutations compared between the durable clinical benefit (DCB) and the no durable benefit (NDB) groups. The median value and the range of the data in each group are shown. **(B)** The comparison of survival rate between gastric cancer patients with high TMB and those with low TMB in our study who received HIPEC treatment. **(C)** The survival analysis between patients with high TMB and those with low TMB from the ICGC database without HIPEC treatment. In **(B, C)**, the overall survival time is shown on the X-axis, and the survival rate with a percentage scale is shown on the Y-axis. **(D)** The number of strong somatic mutations compared between the DCB and the NDB groups in the replication cohort. The median value of each group and the standard deviation showing data variability are shown. **(E)** The comparison of survival rate between gastric cancer patients with high TMB and those with low TMB in the replication cohort who received HIPEC treatment.

We first analyzed the overall TMB of non-synonymous strong somatic mutation with low frequency in a large population database (variant allele frequency <0.01). The TMB was defined as the total number of strong somatic non-synonymous mutations in the coding regions of the human genome. The TMB in the DCB group ranges from 113 to 3,027, with a median of 799.5; and the TMB in the NDB group ranges from 90 to 569, with a median of 278. Therefore, patients with a partial or stable response to HIPEC had a significantly higher TMB than patients with NDB (p = 0.034) (**Figure 1A**). We noticed that there is a subject in the DCB group carrying >3,000 mutations. After removing this sample, the comparison yielded a result of p = 0.07 with marginal significance but still suggests a positive correlation between TMB and response to HIPEC. The K-M survival curves showed significantly better survival in the DCB group [median OS 1,754 vs. 186 days, log-rank p = 0.0001; hazard ratio (HR) = 10.47, 95% confidence interval (CI) ranged from 3.139 to 34.93].

Patients were divided into the high mutation group and the low mutation group based on the median of somatic mutation number of all patients. To measure the effect of TMB on the OS of GC patients, we conducted K-M survival analysis. K-M survival curve showed that patients with high TMB had a significant longer survival time than that of patients with low TMB (HR = 4.82, 95% CI = 1.46–15.95, p = 0.001; **Figure 1B**). In addition, analyzing the data of Chinese gastric cancer patients in the ICGC database, we found no significant association between TMB and patient survival time (p > 0.05; **Figure 1C**). In addition, a previous research with comparable sequencing approach among 262 GC patients after excluding patients who received routine preoperative chemoradiotherapy or biological immunotherapy showed that the prognosis of GC and OS are better among patients with higher TMB than those with lower TMB (28). Thus, the association of high TMB and better response to HIPEC observed in our study is not likely due to the relationship between TMB and prognosis of gastric cancer *per se*. The above evidence suggests an association between high TMB and longer survival for patients who received HIPEC treatment.

We further conducted a replication study. The replication cohort is composed of an independent set of 15 GC samples from patients with similar HIPEC treatment, including 10 patients in the DCB group and 5 patients in the NDB group according to the same criteria. The baseline clinical characteristics of the replication cohort were similar to those of the discovery cohort (**Table 2**). The median TMB was 364.5 in tumors from patients in the DCB group compared to 233 in those in the NDB group (Mann–Whitney p = 0.02) (**Figure 1D**). With the same TMB criteria, the GC samples were classified as high TMB group (n = 7) and low TMB group (n = 8). K-M survival analysis was similarly conducted to measure the effect of TMB on the OS of GC patients in the replication cohort. The results confirmed that patients with high TMB had a significantly longer survival time than that in patients with low TMB (HR = 5.842, 95% CI = 1.179–28.94, p = 0.031; **Figure 1E**).

**Table 2:** The baseline characteristics of patients who received hyperthermic intraperitoneal chemotherapy (HIPEC) in the replication cohort.

	DCB (n = 10)	NDB (n = 5)	p
Age	50.80 ± 10.63	41.24 ± 15.90	0.186
Sex			
Male	8 (80)	1 (80)	0.089
Female	2 (20)	4 (20)	
ECOG			
0–1 score	10 (100)	5 (100)	0.999
2–4 score	0 (0)	0 (0)	
Degree of differentiation			
G1+G2	2 (20)	0 (0)	0.524
G3+G_X_	8 (80)	5 (100)	
P degree			
p1x	7 (80)	1 (20)	0.119
p1-p3	3 (20)	4 (80)	
Ascites			
no	8 (80)	5 (100)	0.524
A small amount	2 (20)	0 (0)	
Number of chemotherapy in half a year			
1–3	4 (40)	3 (60)	0.364
4–6	6 (60)	1 (20)	
≥7	0 (0)	1 (20)	

ECOG, the grade of Eastern Cooperative Oncology Group; DCB, group with durable clinical benefit; NDB, group without durable benefit.

### Single Variant Associated With HIPEC Therapy

We then sought to identify mutations associated with efficacy of HIPEC therapy. The results based on Fisher’s exact test did not suggest any strong somatic mutations surpassing statistical significance, which is likely due to the limitation of a small sample size. Three mutations occurred in either the DCB or the NDB group specifically without carriers in the other group (**Table 3**).

**Table 3:** The three variants that are specifically enriched in the durable clinical benefit (DCB) group or the no durable benefit (NDB) group.

Chr	Pos	Variant	AA alteration	Cases	Controls
11	1092910	NM_002457:exon31:c.G4729A	MUC2:p.G1577S	3	0
19	23158703	NM_001267716:exon4:c.T1436C	ZNF728:p.L479P	3	0
13	103395322	NM_001146197:exon4:c.C7725A	CCDC168: p.N2575K	0	3

### Genes Correlated With HIPEC Treatment

**Figure 2:**
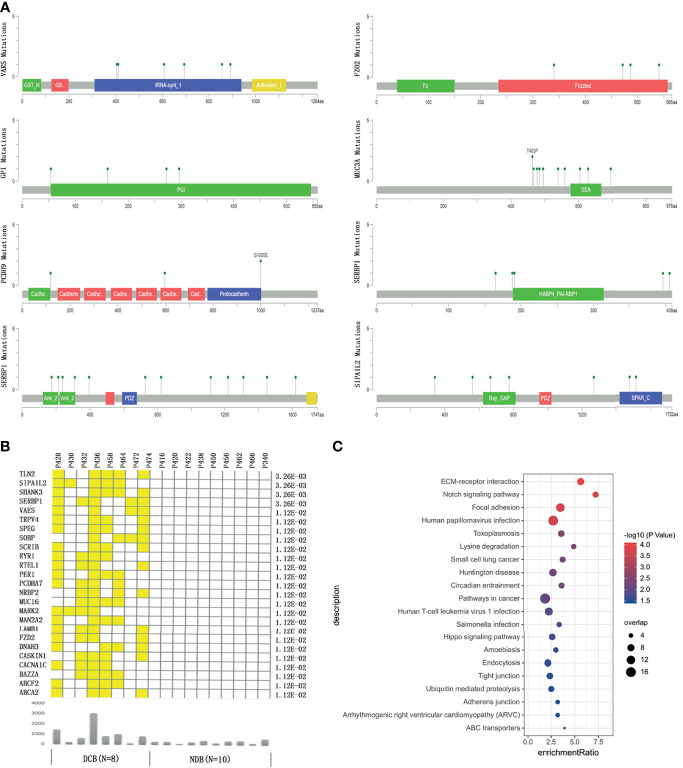
The genes bearing mutations specifically enriched in the durable clinical benefit (DCB) group or no durable benefit (NDB) group. **(A)** The location of the mutations in the top response-associated genes with respect to the structural domains of each protein product encoded by these genes. **(B)** The genes carrying mutations that only occurred in patients in the DCB group but none in the NDB group. The yellow color indicates a patient in the DCB group carrying a mutation in the corresponding gene. The lower panel showed the total number of mutations in each patient. The number on the right shows the p-value of Fisher exact test comparing the number of GC patients carrying mutation(s) in each gene between the DCB and NDB groups. **(C)** The pathways enriched by genes with at least one mutation specifically occurring in patients in the DCB group.

We then focused on identifying genes harboring mutations recurrently and selectively associated with response or resistance to HIPEC. We found that genes *GPI, PCDH9*, and *C21ORF140* harbored mutations in three or more NDB patients but none in the DCB group, and further statistical analyses of collapsing variants to the gene level showed a significant negative correlation with HIPEC response (p < 0.05). On the other hand, a total of 25 genes bearing mutations are recurrently and selectively enriched in the DCB group but none in the NDB group (**Figures 2A, B** **)**. Several of these genes were known to be involved in regulating cancer proliferation, metastasis, or invasion (**Supplementary Table S1**). Analyzing the association between non-synonymous mutations in these genes and OS of Chinese gastric cancer patients in the ICGC database, we did not find any significant association with OS. We checked the distribution of the above enriched single variants and genes in the replication cohort. Gene MUC16 harbored mutations in three patients of the DCB group but none in the NDB group; gene DNAH3 contained mutations in four patients of the DCB group but none in the NDB group; the other genes did not exhibit such enrichment. It is not unexpected for discrepancies to appear at the variant and gene levels between the discovery and replication cohorts, considering the sample size. Thus, studies with larger sample sizes are certainly required to identify and validate predictive markers for HIPEC treatment.

### Gene Set Enrichment Analysis of Significantly Correlated Genes

To identify potential signaling pathways that may contribute to HIPEC sensitivity, we conducted pathway enrichment on 348 genes that bear mutations occurring only in the DCB group but not the NDB group. Among the 134 genes that were mapped to Kyoto Encyclopedia of Genes and Genomes (KEGG) pathways, the pathways of extracellular matrix (ECM)–receptor interaction, Notch signaling pathway, focal expression, and human papillomavirus infection showed significant enrichment (q-value <0.05) (**Figure 2C**).

## Discussion

Though HIPEC has been widely administered to patients of gastric cancer, it has been controversial about its efficacy likely due to tumor heterogeneity; therefore, it is important to identify genomic determinants of response to HIPEC treatment. To our knowledge, this is the first study examining molecular features of HIPEC efficacy using an unbiased high-throughput sequencing approach. Within our discovery cohort and a replication cohort, we found a significant association between high TMB and patients’ beneficial treatment effect.

HIPEC may induce immune response activation. It has been reported that focal thermal ablation of tumor may stimulate a systemic antitumor immune response (31). From the tumor cells and surrounding tissues that were damaged by heat, a variety of immune molecules were released, including cytokines and chemokines. It has been reported that the serum levels of tumor necrosis factor-α (TNFα) and interleukin-1β (IL-1β), IL-6, and IL-8 were elevated after thermal ablation (32–35). In addition, heat shock proteins (HSPs) such as HSP70, which is highly expressed by tumor cells, were detected to be increased following thermal ablation. HSPs play important roles in inhibition of apoptosis inside cells, as well as antigen-presenting and activating dendritic cells and modulating the activity of T-regulatory cells in the microenvironment of tumor cells. HSPs are also involved in other antitumor immune responses (36–39). Increased activation of dendritic cells and decreased activity of T-regulatory cells have been observed in multiple studies (32, 33). Immune cell infiltration was also observed, such as B and T lymphocytes, dendritic cells, neutrophils, natural killer (NK) cells, and macrophages. Such intense immune and inflammatory response occurring after focal thermal ablation may also be able to explain the positive correlation between TMB and response to HIPEC. We hypothesize that tumor cell damage caused by HIPEC results in the release of various intracellular molecules to the tumor microenvironment, and the higher the TMB, the more neo-antigens may be captured by immune cells, therefore leading to better response after treatment.

We identified multiple genes carrying mutations enriched in the DCB or NDB group specifically. The expression level or SNPs in these genes have been reported as being significantly associated with several cancer types. Functional studies showed that these genes have been implicated in key signaling pathways in cancer, such as the proliferation, migration, and invasion of tumor cells, as being summarized in **Supplementary Table S1**. However, due to the limited sample size, the individual variant or gene is not robust enough to be identified as a definitive predictive marker for responses to HIPEC treatment. A larger cohort with more samples needs to be sequenced to validate these findings, and experimental studies need to be carried out to investigate the role of these genes in molecular and cellular responses to HIPEC.

### Limitations

The limitation of our study lies in three aspects. First, the sample size is small; therefore, it is not unexpected that none of the enriched variants at the single-variant level is statistically significant. The results from gene level need to be further examined in studies with larger sample sizes. Second, an independent replication study sampled from other populations is needed to investigate whether TMB that was identified as a predictive mutation feature for HIPEC efficacy can be generalized to other populations. Third, functional studies are warranted to further examine how such a mutational feature contributes to response or resistance to HIPEC treatment. Nevertheless, our study demonstrated that a high mutational burden is significantly associated with better response to HIPEC, which is consistent with previous reports that HIPEC induces immune responses and high TMB confers better immunotherapy efficacy.

### Conclusion

Though limited by the small sample size and single data type at the DNA level, we conducted the first biomarker study of HIPEC treatment to gastric cancer based on unbiased high-throughput sequencing data. We found that TMB is significantly associated with HIPEC efficacy. The genes identified are relevant to the activation and inhibition of signaling pathways in cancer cells. Our results demonstrated that high mutational burden confers a beneficial HIPEC treatment effect, which warrants further confirmation in independent cohorts and functional examination of the underlying mechanism of HIPEC sensitivity. Our study provides insights into the development of mutational features to select patients for effective HIPEC treatment.

## Data Availability Statement

According to national legislation/guidelines, specifically the Administrative Regulations of the People’s Republic of China on Human Genetic Resources (http://www.gov.cn/zhengce/content/2019-06/10/content_5398829.htm, http://english.www.gov.cn/policies/latest_releases/2019/06/10/content_281476708945462.htm), no additional raw data are available at this time. Data of this project can be accessed after an approval application to the China National Genebank (CNGB, https://db.cngb.org/cnsa/). Please refer to https://db.cngb.org/or email CNGBdb@cngb.org for detailed application guidance. The accession code CNP0002628 should be included in the application.

## Ethics Statement

The studies involving human participants were reviewed and approved by the institutional review boards of the Affiliated Cancer Hospital of Guangzhou Medical University. The patients/participants provided their written informed consent to participate in this study.

## Author Contributions

SC and JLi designed and supervised the study. LZ and YT prepared patients’ samples for sequencing and collected clinical data. LZ and XH performed data analysis. The other authors partially participated in the study and helped on data analysis and interpretation. LZ, XH, QX, JLi, and SC wrote the initial article. All authors have read and approved the article.

## Funding

This work was supported by the National Natural Science Foundation of China (No. 81972918, 81771769, and 82172885), Natural Science Foundation of Guangdong Province (No. 2018A030310249, 2020A1515010818, and 2021A1515012392), and Key Clinical Technique of Guangzhou (No. 2019ZD16) and Guangzhou Key Medical Discipline Construction Project Fund.

## Conflict of Interest

Authors KL and YS are employed by Nanjing Geneseeq Technology Inc.

The remaining authors declare that the research was conducted in the absence of any commercial or financial relationships that could be construed as a potential conflict of interest.

## Publisher’s Note

All claims expressed in this article are solely those of the authors and do not necessarily represent those of their affiliated organizations, or those of the publisher, the editors and the reviewers. Any product that may be evaluated in this article, or claim that may be made by its manufacturer, is not guaranteed or endorsed by the publisher.

## References

[1] Van Cutsem E Sagaert X Topal B Haustermans K Prenen H. Gastric Cancer. Lancet (2016) 388:2654–64. doi: 10.1016/S0140-6736(16)30354-3 27156933

[2] Zhu ZG Tang R Yan M Chen J Yang QM Li C. Efficacy and Safety of Intraoperative Peritoneal Hyperthermic Chemotherapy for Advanced Gastric Cancer Patients With Serosal Invasion. A Long-Term Follow-Up Study. Dig Surg (2006) 23:93–102. doi: 10.1159/000093778 16763374

[3] Yang XJ Li Y Yonemura Y. Cytoreductive Surgery Plus Hyperthermic Intraperitoneal Chemotherapy to Treat Gastric Cancer With Ascites and/or Peritoneal Carcinomatosis: Results From a Chinese Center. J Surg Oncol (2010) 101:457–64. doi: 10.1002/jso.21519 20401915

[4] Yang XJ Huang CQ Suo T Mei LJ Yang GL Cheng FL. Cytoreductive Surgery and Hyperthermic Intraperitoneal Chemotherapy Improves Survival of Patients With Peritoneal Carcinomatosis From Gastric Cancer: Final Results of a Phase III Randomized Clinical Trial. Ann Surg Oncol (2011) 18:1575–81. doi: 10.1245/s10434-011-1631-5 PMC3087875 21431408

[5] Brenkman HJF Paeva M Van Hillegersberg R Ruurda JP Haj Mohammad N. Prophylactic Hyperthermic Intraperitoneal Chemotherapy (HIPEC) for Gastric Cancer-A Systematic Review. J Clin Med (2019) 8(10):1685. doi: 10.3390/jcm8101685 PMC6832700 31618869

[6] Koga S Hamazoe R Maeta M Shimizu N Murakami A Wakatsuki T. Prophylactic Therapy for Peritoneal Recurrence of Gastric Cancer by Continuous Hyperthermic Peritoneal Perfusion With Mitomycin C. Cancer (1988) 61:232–7. doi: 10.1002/1097-0142(19880115)61:2<232::AID-CNCR2820610205>3.0.CO;2-U 3121165

[7] Yonemura Y Ninomiya I Kaji M Sugiyama K Fujimura K Sawa T. Prophylaxis With Intraoperative Chemohyperthermia Against Peritoneal Recurrence of Serosal Invasion-Positive Gastric Cancer. World J Surg (1995) 19:450–454; discussion 455. doi: 10.1007/BF00299188 7639005

[8] Hirose K Katayama K Iida A Yamaguchi A Nakagawara G Umeda S. Efficacy of Continuous Hyperthermic Peritoneal Perfusion for the Prophylaxis and Treatment of Peritoneal Metastasis of Advanced Gastric Cancer: Evaluation by Multivariate Regression Analysis. Oncology (1999) 57:106–14. doi: 10.1159/000012016 10461056

[9] Kim JY Bae HS. A Controlled Clinical Study of Serosa-Invasive Gastric Carcinoma Patients Who Underwent Surgery Plus Intraperitoneal Hyperthermo-Chemo-Perfusion (IHCP). Gastric Cancer (2001) 4:27–33. doi: 10.1007/s101200100013 11706624

[10] Kunisaki C Shimada H Nomura M Akiyama H Takahashi M Matsuda G. Lack of Efficacy of Prophylactic Continuous Hyperthermic Peritoneal Perfusion on Subsequent Peritoneal Recurrence and Survival in Patients With Advanced Gastric Cancer. Surgery (2002) 131:521–8. doi: 10.1067/msy.2002.123769 12019405

[11] Kunisaki C Shimada H Nomura M Matsuda G Otsuka Y Ono H. Therapeutic Strategy for Scirrhous Type Gastric Cancer. Hepatogastroenterology (2005) 52:314–8. doi: 10.1159/000084820 15783058

[12] Coccolini F Celotti A Ceresoli M Montori G Marini M Catena F. Hyperthermic Intraperitoneal Chemotherapy (HIPEC) and Neoadjuvant Chemotherapy as Prophylaxis of Peritoneal Carcinosis From Advanced Gastric Cancer-Effects on Overall and Disease Free Survival. J Gastrointest Oncol (2016) 7:523–9. doi: 10.21037/jgo.2016.06.05 PMC4963373 27563441

[13] Liu YW Du Y Chen BA. Effect of Hyperthermic Intraperitoneal Chemotherapy for Gastric Cancer Patients: A Meta-Analysis of the Randomized Controlled Trials. J Int Med Res (2019) 47:5926–36. doi: 10.1177/0300060519882545 PMC7045644 31741406

[14] Granieri S Bonomi A Frassini S Chierici AP Bruno F Paleino S. Prognostic Impact of Cytoreductive Surgery (CRS) With Hyperthermic Intraperitoneal Chemotherapy (HIPEC) in Gastric Cancer Patients: A Meta-Analysis of Randomized Controlled Trials. Eur J Surg Oncol (2021) 47:2757–67. doi: 10.1016/j.ejso.2021.05.016 34001385

[15] Yap DRY Wong JSM Tan QX Tan JW Chia CS Ong CJ. Effect of HIPEC on Peritoneal Recurrence in Peritoneal Metastasis Treated With Cytoreductive Surgery: A Systematic Review. Front Oncol (2021) 11:795390. doi: 10.3389/fonc.2021.795390 34926311 PMC8678115

[16] Li Y Yu Y Liu Y. Report on the 9(Th) International Congress on Peritoneal Surface Malignancies. Cancer Biol Med (2014) 11:281–4. doi: 10.7497/j.issn.2095-3941.2014.04.008 PMC4296089 25610715

[17] Van Driel WJ Koole SN Sikorska K Schagen Van Leeuwen JH Schreuder HWR Hermans RHM. Hyperthermic Intraperitoneal Chemotherapy in Ovarian Cancer. N Engl J Med (2018) 378:230–40. doi: 10.1056/NEJMoa1708618 29342393

[18] Kwakman R De Cuba EM De Winter JP De Hingh IH Delis-Van Diemen PM Tijssen M. Tailoring Heated Intraperitoneal Mitomycin C for Peritoneal Metastases Originating From Colorectal Carcinoma: A Translational Approach to Improve Survival. Br J Cancer (2015) 112:851–6. doi: 10.1038/bjc.2015.18 PMC4453952 25668003

[19] Sluiter NR De Cuba EM Kwakman R Meijerink WJ Delis-Van Diemen PM Coupe VM. Versican and Vascular Endothelial Growth Factor Expression Levels in Peritoneal Metastases From Colorectal Cancer Are Associated With Survival After Cytoreductive Surgery and Hyperthermic Intraperitoneal Chemotherapy. Clin Exp Metastasis (2016) 33:297–307. doi: 10.1007/s10585-016-9779-9 26873137 PMC4799792

[20] Shannon NB Tan JW Tan HL Wang W Chen Y Lim HJ. A Set of Molecular Markers Predicts Chemosensitivity to Mitomycin-C Following Cytoreductive Surgery and Hyperthermic Intraperitoneal Chemotherapy for Colorectal Peritoneal Metastasis. Sci Rep (2019) 9:10572. doi: 10.1038/s41598-019-46819-z 31332257 PMC6646658

[21] Hulshof EC Lurvink RJ Caserta N De Hingh I Van Wezel T Bohringer S. Identification of Pharmacogenetic Biomarkers for Efficacy of Cytoreductive Surgery Plus Hyperthermic Intraperitoneal Mitomycin C in Patients With Colorectal Peritoneal Metastases. Eur J Surg Oncol (2020) 46:1925–31. doi: 10.1016/j.ejso.2020.04.019 32354538

[22] Bijelic L Sugarbaker PH. The Role of Intraperitoneal Chemotherapy in the Treatment of Patients With Advanced Gastric Cancer. Ann Ital Chir (2012) 83:224–31. 22595734

[23] Li H Durbin R. Fast and Accurate Long-Read Alignment With Burrows-Wheeler Transform. Bioinformatics (2010) 26:589–95. doi: 10.1093/bioinformatics/btp698 PMC2828108 20080505

[24] Li H Durbin R. Fast and Accurate Short Read Alignment With Burrows-Wheeler Transform. Bioinformatics (2009) 25:1754–60. doi: 10.1093/bioinformatics/btp324 PMC2705234 19451168

[25] Lai Z Markovets A Ahdesmaki M Chapman B Hofmann O Mcewen R. VarDict: A Novel and Versatile Variant Caller for Next-Generation Sequencing in Cancer Research. Nucleic Acids Res (2016) 44:e108. doi: 10.1093/nar/gkw227 27060149 PMC4914105

[26] Wang K Li M Hakonarson H. ANNOVAR: Functional Annotation of Genetic Variants From High-Throughput Sequencing Data. Nucleic Acids Res (2010) 38:e164. doi: 10.1093/nar/gkq603 20601685 PMC2938201

[27] Wang F Wei XL Wang FH Xu N Shen L Dai GH. Safety, Efficacy and Tumor Mutational Burden as a Biomarker of Overall Survival Benefit in Chemo-Refractory Gastric Cancer Treated With Toripalimab, a PD-1 Antibody in Phase Ib/II Clinical Trial NCT02915432. Ann Oncol (2019) 30:1479–86. doi: 10.1093/annonc/mdz197 PMC6771223 31236579

[28] He X Yu M Wang X Chen J Li X. Analysis of Threshold Change of Tumor Mutation Burden in Gastric Cancer. J Oncol (2021) 2021:3374939. doi: 10.1155/2021/3374939 34335754 PMC8321718

[29] Zhan X Hu Y Li B Abecasis GR Liu DJ. RVTESTS: An Efficient and Comprehensive Tool for Rare Variant Association Analysis Using Sequence Data. Bioinformatics (2016) 32:1423–6. doi: 10.1093/bioinformatics/btw079 PMC4848408 27153000

[30] Liao Y Wang J Jaehnig EJ Shi Z Zhang B. WebGestalt 2019: Gene Set Analysis Toolkit With Revamped UIs and APIs. Nucleic Acids Res (2019) 47:W199–205. doi: 10.1093/nar/gkz401 PMC6602449 31114916

[31] Chu KF Dupuy DE. Thermal Ablation of Tumours: Biological Mechanisms and Advances in Therapy. Nat Rev Cancer (2014) 14:199–208. doi: 10.1038/nrc3672 24561446

[32] Ali MY Grimm CF Ritter M Mohr L Allgaier HP Weth R. Activation of Dendritic Cells by Local Ablation of Hepatocellular Carcinoma. J Hepatol (2005) 43:817–22. doi: 10.1016/j.jhep.2005.04.016 16087270

[33] Fietta AM Morosini M Passadore I Cascina A Draghi P Dore R. Systemic Inflammatory Response and Downmodulation of Peripheral CD25+Foxp3+ T-Regulatory Cells in Patients Undergoing Radiofrequency Thermal Ablation for Lung Cancer. Hum Immunol (2009) 70:477–86. doi: 10.1016/j.humimm.2009.03.012 19332094

[34] Ahmad F Gravante G Bhardwaj N Strickland A Basit R West K. Changes in Interleukin-1beta and 6 After Hepatic Microwave Tissue Ablation Compared With Radiofrequency, Cryotherapy and Surgical Resections. Am J Surg (2010) 200:500–6. doi: 10.1016/j.amjsurg.2009.12.025 20887844

[35] Erinjeri JP Thomas CT Samoilia A Fleisher M Gonen M Sofocleous CT. Image-Guided Thermal Ablation of Tumors Increases the Plasma Level of Interleukin-6 and Interleukin-10. J Vasc Interv Radiol (2013) 24:1105–12. doi: 10.1016/j.jvir.2013.02.015 PMC4167629 23582441

[36] Srivastava P. Interaction of Heat Shock Proteins With Peptides and Antigen Presenting Cells: Chaperoning of the Innate and Adaptive Immune Responses. Annu Rev Immunol (2002) 20:395–425. doi: 10.1146/annurev.immunol.20.100301.064801 11861608

[37] Schueller G Kettenbach J Sedivy R Stift A Friedl J Gnant M. Heat Shock Protein Expression Induced by Percutaneous Radiofrequency Ablation of Hepatocellular Carcinoma In Vivo. Int J Oncol (2004) 24:609–13. doi: 10.3892/ijo.24.3.609 14767545

[38] Rai R Richardson C Flecknell P Robertson H Burt A Manas DM. Study of Apoptosis and Heat Shock Protein (HSP) Expression in Hepatocytes Following Radiofrequency Ablation (RFA). J Surg Res (2005) 129:147–51. doi: 10.1016/j.jss.2005.03.020 15975593

[39] Teng LS Jin KT Han N Cao J. Radiofrequency Ablation, Heat Shock Protein 70 and Potential Anti-Tumor Immunity in Hepatic and Pancreatic Cancers: A Minireview. Hepatobil Pancreat Dis Int (2010) 9:361–5. 20688598

