# Supplementary material for: Tumor Mutational Burden Associated With Response to Hyperthermic Intraperitoneal Chemotherapy
Source: Front Oncol. 2022 Mar 8;12:796263. doi: 10.3389/fonc.2022.796263 (PMC8958003; doi:10.3389/fonc.2022.796263)
Supplement: Supplementary file 1 [file Table_1.pdf]

Table S1. The known molecular function and role of top associated genes in cancer.

Gene symbol	Chr	Start	End	Gene Name	Molecular function	Roles in tumors (PMID)
SIPA1L2	1	232,533,711	232,697,304	Signal Induced Proliferation Associated 1 Like 2	1. Belongs to the the signal-induced proliferation-associated 1 like family;<br>2. contain a GTPase activating domain, a PDZ domain and a coiled-coil domain with a leucine zipper;<br>3. may have GTPase activator activity	1. Associated with unfavorable prognosis in cancers (31156702)
SHANK3	22	51,112,843	51,171,726	SH3 And Multiple Ankyrin Repeat Domains 3	1. multidomain scaffold proteins of the postsynaptic density;<br>2. recruits proteins to the actin cytoskeleton and G-protein-coupled signaling pathways	SHANK3 has been shown to be mutated and rearranged upon chemotherapy exposure, which contributes to resistance to chemotherapy (30760869).
SERBP1	1	67,873,493	67,896,123	SERPINE1 MRNA Binding Protein 1	1. regulate mRNA stability<br>2. involved in PML-nuclear bodies formation<br>3. important for ATR-dependent double strand break signaling<br>4. plays a role in homologous recombination-mediated DNA repair during S phase	1. over-expressed in multiple cancer types, including ovarian cancer, breast cancer, non-small cell lung carcinoma, glioblastoma and acute lymphoblastic leukemia (20124481, 23236990, 12960427, 17698176, 23737970)<br>2. regulate the stability of PAI-1 mRNA and its overexpression is correlated with favourable prognosis of ovarian cancer (23236990).<br>3. Overexpressed in glioblastoma and correlates with poor patient survival and poor response to chemo- and

						radiotherapy by affecting cancer metabolism and epigenetic regulation of H3K27me3 sites (32762776)<br>4. included in the ribosomes of human cancer cells (30039520).<br>5. overexpression was significantly associated with tumor differentiation, lymphatic metastasis and Ki67 expression in esophageal squamous carcinoma cells. Knocking down SREBP1 inhibited the proliferation, migration and invasion of ESCC cells through Wnt/β‑catenin signaling pathway (32765792).<br>6. is targeted by miR-218, inhibiting the proliferation, migration, and EMT (28369267).
PCDH9	13	66,876,966	67,804,468	Protocadherin 9	1. have cadherin domains and a transmembrane region;<br>2. mediate cell adhesion with calcium in neural tissues;<br>3. function in signaling at neuronal synaptic junctions	1. PCDH9 affected growth, proliferation and tumorigenic potential of hepatocellular carcinoma. PCDH9 induced tumor cell arrest at G0/G1 phase and its promoter is often methylated, and thus function as a tumor suppressor in hepatocellular carcinoma (28791409).<br>2. inhibits epithelial–mesenchymal transition and cell migration through suppressing Akt and Erks and activating GSK-3β. in hepatocellular carcinoma (25172662).<br>3. Low expression of PCDH9 is associated with poor prognosis for patients with glioma (22300792).<br>4. Regulates glioma cell apoptosis and invasion by upregulating Bax protein expression, but downregulating Bcl-2 and cyclin D1 expression, causing caused G0/G1 cell cycle arrest (24214103).<br>5. miR-200a-3p bound to 3’ UTR of PCDH9 and

						down-regulates PCDH9 expression and thus stimulated the malignant behaviors of ovarian cancer cells (31632082)
TLN2				Talin 2	1. Functions in actin-filament assembly;<br>2. A player in Ras signaling pathway and focal adhesion pathways<br>3. Regulate spreading and migration of various cell types	1. TLN2 is overexpressed in breast cancer tissues. TLN2 regulates breast cancer cell migration and invasion through apoptotic process (29928413).<br>2. TLN2 regulates migration, invasion and metastasis in tumor cells by affecting traction force generation, focal adhesion dynamics and invadopodium formation (30091412)<br>3. lnc-TLN2-4:1 is able to suppress the migration and invasion of gastric cancer cells but does not have an impact on cell proliferation (.32256587)
